# Motivational Profiling of League of Legends Players

**DOI:** 10.3389/fpsyg.2020.01307

**Published:** 2020-07-22

**Authors:** Florian Brühlmann, Philipp Baumgartner, Günter Wallner, Simone Kriglstein, Elisa D. Mekler

**Affiliations:** ^1^Human-Computer Interaction Research Group, Department of Psychology, Center for General Psychology and Methodology, University of Basel, Basel, Switzerland; ^2^Department of Industrial Design, Eindhoven University of Technology, Eindhoven, Netherlands; ^3^Faculty of Business and IT, Ontario Tech University, Oshawa, ON, Canada; ^4^Institute of Art and Technology, University of Applied Arts Vienna, Vienna, Austria; ^5^AIT Austrian Institute of Technology GmbH, Vienna, Austria; ^6^Faculty of Computer Science, University of Vienna, Vienna, Austria; ^7^Department of Computer Science, Aalto University, Espoo, Finland

**Keywords:** motivation, MOBA, game analytics, self-determination theory, latent profile analysis

## Abstract

Player motivation is a key research area within games research, with the aim of understanding how the motivation of players is related to their experience and behavior in the game. We present the results of a cross-sectional study with data from 750 players of *League of Legends*, a popular Multiplayer Online Battle Arena game. Based on the motivational regulations posited by Self-Determination Theory and Latent Profile Analysis, we identify four distinct motivational profiles, which differ with regards to player experience and, to a lesser extent, in-game behavior. While the more self-determined profiles “Intrinsic” and “Autonomous” report mainly positive experience-related outcomes, a considerable part of the player base does not. Players of the “Amotivated” and “External” profile derive less enjoyment, experience more negative affect and tension, and score lower on vitality, indicating game engagement that is potentially detrimental to players' well-being. With regards to game metrics, minor differences in the rate of assists in unranked matches and performance indicators were observed between profiles. This strengthens the notion that differences in experiences are not necessarily reflected in differences in behavioral game metrics. Our findings provide insights into the interplay of player motivation, experience, and in-game behavior, contributing to a more nuanced understanding of player-computer interaction.

## 1. Introduction

For many people, playing games is one of the most rewarding and motivating activities. In turn, people's motivation for playing games shapes their player experience and in-game behavior (e.g., Yee et al., 2012; Canossa et al., 2013; Schaekermann et al., 2017; Melhart et al., 2019), as well as their well-being (Przybylski et al., 2009; Vella et al., 2013; Perry et al., 2018). However, while concepts from motivational psychology, particularly Self-Determination Theory (SDT, Deci and Ryan, 2000), commonly inform research on player experience (Tyack and Mekler, 2020) and game analytics (e.g., Canossa et al., 2013; Melhart et al., 2019), the notion of motivational regulation (Deci and Ryan, 2000)has received limited attention in the context of games (Tyack and Mekler, 2020). This is an unfortunate gap in our understanding of the player-computer interaction, as motivational regulations have been found to determine to what extent people experience positive emotions and need satisfaction, as well as how persistently they engage in a behavior (Neys et al., 2014). Motivational regulations describe an underlying regulatory process of people's motivation, which determine the quality of their behavior, the extent of need satisfaction they experience, and the impact of these behaviors on their well-being (Deci and Ryan, 2000). Multiplayer Online Battle Arena (MOBA) games pose a particularly intriguing case. They enjoy enduring popularity, with a player base ranging in the millions, despite often affording a range of negative experiences (Johnson et al., 2015; Tyack et al., 2016). Specifically, MOBA players report decreased autonomy and increased frustration (Johnson et al., 2015), counter to SDT-based notions of positive player experience. Moreover, they afford complex, sometimes uncomfortable, social interactions amidst a highly competitive gaming environment. Considering players' underlying motivational regulations may hence provide a better understanding of the interplay of player experience and in-game behavior in MOBA games.

Identifying motivational profiles may enable us to study similarities between players and to highlight differences in experience, well-being, and behavior between these profiles. In that sense, this paper provides researchers and game designers with enhanced knowledge to better discern differing motivations and with it, experiences of their player basis. Building upon previous workon player profiling (e.g., Drachen et al., 2014; Chen et al., 2017; Nascimento Junior et al., 2017; Schaekermann et al., 2017), we present the results of a cross-sectional study with self-report and behavioral data from 750 players of *League of Legends* (*LoL*, Riot Games, 2009), a popular MOBA game. Drawing from work on SDT-based motivational profiling (Pastor et al., 2007; Howard et al., 2016; Wang et al., 2017; Gustafsson et al., 2018), we identify four distinct motivational player profiles (i.e., Amotivated, External, Intrinsic, and Autonomous) and compare these in terms of player experience and in-game behavior. We provide empirical evidence of the relation between motivational regulation and player experience. Specifically, we show that despite overall high intrinsic motivation, players can be categorized into distinct motivational profiles, which affect their quality of experience. Intrinsically and Autonomously motivated player profiles report consistently more positive player experiences, as evidenced by high scores on enjoyment, need satisfaction, and harmonious passion. In contrast, already slight increases in amotivation and external motivation were related to reduced enjoyment, more tension, and less harmonious passion, indicating game engagement that is potentially less conducive to players' well-being (Vella et al., 2013; Johnson et al., 2016). These findings extend our understanding of the role of motivation for the player-computer interaction, as well as provide context for conflicting results regarding the player experience of MOBA games (Johnson et al., 2015; Tyack et al., 2016). Second, we investigate how player motivation relates to in-game behavior, where we observe only a few clear-cut differences between motivational profiles. As such, our findings showcase that even when little to no behavioral differences are apparent, motivational regulations clearly color the quality of player experience.

## 2. Related Work

In the following section, we first review research around the interplay of player motivation, experience, and in-game behavior, after which we outline key motivational regulations posited by SDT and research on motivational profiling. Finally, we focus on the unique properties of MOBA games.

### 2.1. Player Motivation

Player motivation is a central research area in player-computer interaction, where the goal is to gain a better understanding of how motivational factors shape players' experience and behavior.

#### 2.1.1. Motivation and Player Experience

Motivation is widely considered a key determinant of players' gaming experiences and preferences. Early works primarily linked motivation to typologies of player preferences and were not grounded in any established psychological frameworks or theories of human motivation. Bartle (1996), for instance, identified four distinct player “types” with varying play preferences in Multi-User Dungeon games. Similarly, Yee (2006) identified achievement, immersion, and social aspects of gameplay as key motivators for why people find playing online games appealing.

More recently, a growing body of player motivation research has emerged around Self-Determination Theory (SDT), a major psychological theory of human motivation (Deci and Ryan, 2000; Ryan and Deci, 2000). Notably, Ryan et al. (2006) criticized Yee's player motivation typology for focusing only on game content, rather than considering universal personal factors that generalize across a variety of players and game genres. Instead, they demonstrated in a series of studies that satisfaction of innate psychological needs for autonomy, competence, and relatedness, predict game enjoyment and future play across a variety of game genres. Indeed, this relation between psychological need satisfaction and positive player experience has been repeatedly demonstrated across several studies (e.g., Vella et al., 2013; Neys et al., 2014; Johnson et al., 2015, see also Tyack and Mekler, 2020 for a recent overview). Moreover, need satisfaction has also been linked to increased time spent playing (Johnson et al., 2016).

#### 2.1.2. Motivation and In-Game Behavior

Digital games motivate a variety of goal-directed behaviors (Przybylski et al., 2010), which may be reflected in players' in-game behavior (Schaekermann et al., 2017). As such, a growing body of research has emerged around detecting player motivation profiles from game metrics. Specifically, game analytics provide detailed and granular insights into players' in-game behavior to identify hot spots or problem areas (e.g., Drachen and Canossa, 2009; Wallner et al., 2014). Bauckhage et al. (2012), for example, investigated behavioral telemetry data from five different games to understand how players engaged with these games over a longer time period. Similarly, Harpstead et al. (2015) presented an approach for creating engagement profiles of game players. In the context of massively multiplayer online role-playing games, Feng et al. (2007) analyzed long-term player workloads and behavior in *EVE Online* (CCP, 2003). Suznjevic et al. (2011) identified categories of player actions in *World of Warcraft* (Blizzard Entertainment, 2004), which formed the basis for creating a player behavior model and combined it with network traffic models of the action categories.

However, while game analytics provide insight into players' in-game behavior, that is, *what* they are doing when playing, consideration of motivational frameworks may help contextualize *why* players behave in such a way (Hazan, 2013). Other works therefore attempted to link pre-defined motivational categories to in-game behavior. Yee et al. (2012), for instance, found that players' in-game behavior in *World of Warcraft* (Blizzard Entertainment, 2004) was to some extent predictive of their motivation (i.e., the aforementioned motives for immersion, achievement, and social interaction, Yee, 2006). Players motivated by achievement, for example, were more likely to engage in dungeoneering and Player vs. Player battles. In another study, Schaekermann et al. (2017) correlated self-reported player curiosity scores with in-game behavioral metrics in *Destiny* (Bungie, Inc., 2014), with curiosity considered a motivational driver for playing games. Among their results, they found that social curiosity was positively correlated to players' tendency toward exploratory behavior. Finally, some studies applied combined motivational psychology, data analysis, and machine learning techniques to better predict player engagement. Canossa et al. (2013), for example, investigated bivariate correlations and applied multiple supervised learning methods to identify relationships between in-game behavior in *Minecraft* (Mojang, 2011) and motivational factors, as measured by the Reiss Motivation Profiler (Reiss and Havercamp, 1998). Melhart et al. (2019), in contrast, employed support vector machines to predict motivation in *Tom Clancy's: The Division* (Massive Entertainment, 2016) based on game metrics. They found that both linear and non-linear models successfully predicted motivation with an average accuracy of 65.89 and 75.62% respectively. Notably, motivation was measured by the Ubisoft Perceived Experience Questionnaire (Azadvar and Canossa, 2018), a proxy for psychological need satisfaction in games, as posited by SDT (Ryan et al., 2006). However, correlations between the self-reported measures and game metrics remained weak.

### 2.2. Motivational Regulation

Organismic Integration Theory (OIT), a mini–theory of SDT, differentiates six types of motivational regulations (Deci and Ryan, 2000; Ryan and Deci, 2000). According to OIT, the underlying regulation of people's motivation determine the quality of their behavior, the extent of need satisfaction they experience and the consequences of these behaviors for their well-being (Deci and Ryan, 2000).

**Figure 1:**
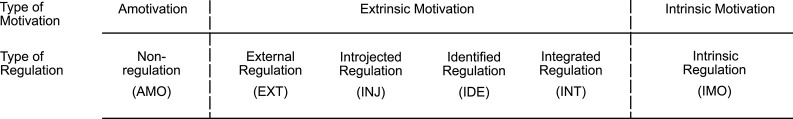
The six types of motivational regulation as posited by Self-Determination Theory. Ranging from the least self-determined (amotivation) to the most self-determined regulation (intrinsic motivation). Figure adapted from Deci and Ryan (2002), p. 16.

As depicted in Figure 1, these motivational regulations range on a spectrum from non-self-determined (amotivation) to fully self-determined (intrinsic motivation). Set in context, need satisfaction is an outcome of pursuing an activity (Deci and Ryan, 2000), while the degree to which an activity (e.g., playing a game) supports need satisfaction is determined by the underlying motivational regulation (e.g., *why* an activity is being pursued). Consequences (e.g., decreased need satisfaction) are more negative, the less self-determined the motivation for pursuing that activity is (Deci and Ryan, 2002). Specifically, OIT distinguishes three types of motivation: (1) Amotivation describes a lack or absence of motivation, hence being the least self-determined form of motivational regulation. (2) Extrinsic motivation refers to activity pursued for a separable outcome. More precisely, SDT distinguishes different types of extrinsic motivation comprised of four types of regulations: *external regulation* (EXT), *introjected regulation* (INJ), *identified regulation* (IDE), and *integrated regulation* (INT). EXT is the least self-determined form of extrinsic motivation and occurs in situations where people act to obtain a reward or avoid punishment (e.g., other players would pressure me if I perform badly at *League of Legends*). INJ regulation has been partially internalized, but not truly accepted as one's own. Such behaviors are pursued to avoid guilt or shame or to achieve feelings of self-worth or approval. IDE follows from the conscious valuing of an activity as personally important, rendering the pursuit of such an activity more self-determined. INT results when an activity is congruent with personally endorsed values and goals, and thus forms the most self-determined regulation among extrinsic motivations. Finally, (3) intrinsic motivation refers to an activity being pursued for its own sake, because it is experienced as enjoyable and interesting (Deci and Ryan, 2000).

#### 2.2.1. Motivational Regulation in Human-Computer Interaction and Games

Motivational regulations, as posited by OIT, have also been explored within Human-Computer Interaction (HCI) and games research. In the context of general technology use, for instance, Brühlmann et al. (2018) developed and validated the User Motivation Inventory (UMI), an instrument that covers the whole spectrum of motivational regulation. Specifically, Brühlmann et al. (2018) found that respondents who reported higher levels of amotivation and scored lower on more self-determined regulations (IDE, INT) and intrinsic motivation, were more likely to consider to stop using a device. In contrast, participants scoring high on more self-determined and autonomous motivations reported more positive user experiences. Similarly, Peters et al. (2018) applied OIT to create a model that describes and predicts the impact of technologies on technology adoption, engagement and well-being. Hence, a better understanding of users' motivational regulations may help detect and prevent user churn, as well as identify potential negative effects of technology use on well-being.

The notions of need satisfaction and intrinsic motivation are also prevalent in player-computer interaction research (Tyack and Mekler, 2020). However, OIT has received relatively little attention (Tyack and Mekler, 2020). A few works have employed the Situational Motivation Scale (SIMS, Guay et al., 2000), but report no results (Alexandrovsky et al., 2019; Johanson et al., 2019). Birk and Mandryk (2018) used the SIMS to assess whether customization affected participants' motivation and behavior in a game-like self-improvement program taking place over 3 weeks. Curiously, they found that while customization resulted in significantly less attrition and more login counts, participants' self-reported motivation remained unaffected. Finally, Lafrenière et al. (2012) developed the Gaming Motivation Scale (GAMS), a questionnaire that assesses all six motivational regulations, specifically in the context of gaming.

Of particular interest to the present work, OIT has also been applied to study the player experience and gaming persistence of hardcore, heavy, and more casual players (Neys et al., 2014). Self-identified hardcore gamers reported the highest degree of intrinsic motivation and identified regulation, but also slightly elevated levels of external regulation, compared to heavy and casual gamers. Curiously, while also scoring high on intrinsic motivation and identified regulation, casual gamers scored highest on amotivation. With regards to playing persistence, immediate enjoyment was most predictive, but intrinsic motivation and external regulation were also significantly associated with increased persistence.

#### 2.2.2. Motivational Regulation Profiles

More recently, works have drawn from OIT and attempted to profile people according to their motivational regulations. Gustafsson et al. (2018) explored the link between elite athletes' motivational profiles and burnout. Using Latent Profile Analysis (LPA), they identified five profiles with distinct patterns of motivational regulations. Athletes with high levels of amotivation as well as moderately controlled regulation showed higher burnout risk when compared to other profiles from the LPA. The quality of athletes' motivations might therefore be an important factor in protecting them from negative outcomes related to their health, performance and well-being. In the workplace setting, Howard et al. (2016) identified four motivational profiles of two samples of employees from different countries. They found that autonomous forms of motivation support positive workplace-related outcomes, such as performance and well-being. In another study, Wang et al. (2017) used LPA to identify four motivational profiles in secondary school students. Results showed that students in the highly self-determined motivational profile reported more effort, higher competence, value, and time spent on math beyond homework, when compared to the other profiles. In Pastor et al. (2007), LPA was used to classify college students into different goal orientation profiles using 2-, 3-, and 4-factor conceptualizations of goal orientation. The main goal was to show the advantages of LPA over other clustering methods. By using LPA, they were able to apply stricter criteria when deciding upon the final cluster solutions, represent students' cluster membership partially, and classify students from a different sample into clusters. This would not have been possible to the same extent with multiple regression or cluster analysis. Therefore, a person-centered approach to the study of motivational regulations seems promising.

### 2.3. MOBA Games

Multiplayer online battle arena (MOBA) games have been extremely popular throughout the years and are among the most profitable games on the market^1^. Thus, it comes to little surprise that a growing body of research has emerged around MOBA players' experience and behavior to better understand what keeps them motivated to play (see Mora-Cantallops and Sicilia, 2018, for a recent overview).

Johnson et al. (2015), for instance, found that compared to other genres, MOBA players report increased frustration and a reduced sense of autonomy. The authors hypothesize that this may be due to the intense competition with others. Relatedly, Kou et al. (2018) identified streakiness, i.e., whether players had winning or losing streaks—as crucial to player retention and experience of League of Legends, potentially because it impacts players' sense of competence Kou et al. (2018). Indeed, a common reason to quit playing MOBAs is that players simply do not experience them as fun anymore (Tyack et al., 2016).

Besides their competitive nature, MOBAs are also known for the complex social interactions they afford, with toxic player behavior among the major sources of negative experiences (Kwak and Blackburn, 2014; Kwak et al., 2015; Tyack et al., 2016). Tyack et al. (2016), for instance, identified deviant behavior from teammates as a reason to abandon playing MOBA games, although most players ultimately quit due to reasons unrelated to the game. In contrast, the opportunity to play with friends is a key motivator to start and keep playing MOBAs. However, despite this growing body of work around player churn and retention, none of the aforementioned studies have examined how players' experience relate to their in-game behavior.

With regards to players' in-game behavior, works have attempted to detect patterns in combat tactics of winning teams (Yang et al., 2014) based on the game data from *Dota 2* (Valve Corporation, 2013), analyzed professional and public matches for classifying playstyles (Gao et al., 2013), as well as classified player behavior in order to identify roles within player teams (Eggert et al., 2015). However, none of these works have considered players' motivation to engage with MOBAs. A notable exception is the work by Kahn et al. (2015), who developed a typology of player motives, similar to the work by Yee (2006), Yee et al. (2012). They validated their typology on a sample of over 18,000 League of Legends players and correlated the questionnaire with various game metrics. The motive to socialize was correlated with the average percentage of teammates that players already knew, whereas the completionist motivation was correlated with the number of different champions played. Finally, competitiveness was positively correlated with the number of kills and killing sprees. However, Kahn et al. (2015) did not explore how these motives relate to players' experience, nor is their typology grounded in any established framework of human motivation.

## 3. Methods

The aim of this study was to explore how players' underlying motivational regulations relate to their experience and in-game behavior in a MOBA game. In contrast to previous research on predicting motivation from in-game metrics (Melhart et al., 2019), we present a novel, theory-driven approach for detecting motivational profiles, and compare these in terms of player experience and in-game behavior.

### 3.1. League of Legends

*League of Legends* (*LoL*) (Riot Games, 2009) is a MOBA game where players take on the role of *summoners* that control a single character (i.e., champion). Two teams of usually three or five players compete against each other. The two teams start on opposite sides of a map near a main building called *Nexus*. The goal of the game is to destroy the enemy's Nexus. The Nexus is defended by the enemy team, computer-controlled units (so-called “minions”) and towers. The minions are sent in the direction of the enemy main building and follow certain paths (so-called “lanes”) and attack close enemies. By killing minions, monsters, enemy champions, and destroying enemy towers, the player's own champion gains experience, i.e., they reach a higher level where new abilities can be unlocked or improved. These abilities are determined by the respective champion and are not freely selectable. In addition, the player who delivers the final deathblow to an enemy unit will receive a certain amount of gold. This gold can be used to purchase special items for the champion in the base, which improve various attributes (such as attack damage) or otherwise have positive effects. At the time of writing, there were a total of three maps with different game modes available. Among others, *LoL* offers the game modes “ranked” and “unranked” matches. Ranked matches are recorded in a central ranking system. Upon winning, players ascend in the rankings, and move down when they lose. Ranked games resemble unranked games but require a summoner level of 30 and a minimum of 20 champions to participate.

We chose to focus on *LoL*, because it is to date one of the most played games in the world^2^. Moreover, LoL is known to afford complex, sometimes negative social interactions (e.g., Kwak and Blackburn, 2014), and is among the most studied games in the MOBA research literature (Mora-Cantallops and Sicilia, 2018). Because of this complexity and the large player base, we expected that a variety of motivational regulations were present. Another advantage of *LoL* is the availability of a public Application Programming Interface (API), which allowed us to collect activity data to investigate player in-game behavior.

### 3.2. Participants

The survey was advertised on the *League of Legends* subreddit on the American social news aggregation website reddit.com. A total of 2,056 people started the survey, of which 877 completed the survey. Forty-four participants were excluded for not passing the instructed response item (*This is a verification Item. Please choose “Strongly disagree”*) (Brühlmann et al., 2020). We also conducted a longstring analysis to detect repeated answering schemes among the User Motivation Inventory (UMI) items (as in Brühlmann et al., 2018). However, no additional cases were flagged for exclusion through this procedure. Of the remaining 833 participants, 83 did not provide valid summoner names or showed incomplete data sets and were subsequently removed. After data cleaning, 750 participants were included in the analysis. Forty-five participants were women (6 percent), nine participants identified as non-binary and 12 preferred not to specify their gender. Participants' age ranged between 18 and 65 years (*M* = 21.5 years, *SD* = 4.05 years). In total, participants had played between seven and 5,012 matches (*M* = 1577.3 matches, *SD* = 860.5), with summoner levels ranging from 30 to 234 (*M* = 90.8, *SD* = 31.3).

### 3.3. Procedure

Upon clicking the survey link, participants were introduced to the study. After providing consent, participants were asked to provide basic demographic information (gender, age, experience with MOBAs, experience with playing *LoL*), their summoner name (i.e., the name the player is known in the game) and player region. The latter two were used to collect in–game data through the API made available by Riot Games (Riot Games, 2018). Participants then rated their motivation for playing *LoL* and answered a variety of player experience measures (see section 3.4). The individual measures were presented in a constant sequence, but the order of items was randomized for each measure. Finally, participants were given the option to comment on the survey and asked to indicate whether they had answered questions conscientiously. Participants did not receive any compensation for completing the survey, but were presented with a *LoL* “Player-Style” badge as a reward, similar to how previous work (Schaekermann et al., 2017) provided Brainhex (Nacke et al., 2014) badges upon survey completion. On average, the survey took 12 min to complete.

### 3.4. Measures

We collected subjective self-report measures and behavioral game metrics. All self-report measures consisted of 7-point Likert scales ranging from strongly disagree (1) to strongly agree (7), unless noted otherwise. Descriptive statistics and reliability scores (Cronbach's α and hierarchical ω) for each measure are depicted in Table 1.

**Table 1:** Means (M), standard deviations (SD), medians (Mdn), Cronbach's α, and hierarchical omega (ω) for all self-report measures over all participants (*N* = 750) and for each profile.

	**M (SD)**	**Mdn**	**α**	**ω**	**Amotivated ( 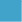 )**	**External ( 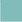 )**	**Intrinsic ( 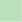 )**	**Autonomous ( 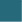 )**	**No. of items**
					***n* = 220**	***n* = 329**	***n* = 90**	***n* = 111**	
**UMI**									**18**
IMO	5.31 (1.39)	5.67	0.86	0.88	5.11 (1.40)	4.97 (1.51)	6.34 (0.55)	5.92 (0.70)	3
INT	3.08 (1.48)	3.00	0.80	0.80	2.84 (1.47)	3.19 (1.49)	2.98 (1.59)	3.29 (1.28)	3
IDE	3.38 (1.39)	3.33	0.70	0.71	3.15 (1.40)	3.50 (1.42)	3.23 (1.44)	3.57 (1.16)	3
INJ	2.35 (1.55)	1.67	0.81	0.81	1.70 (0.80)	3.33 (1.75)	1.03 (0.10)	1.78 (0.64)	3
EXT	1.88 (1.22)	1.33	0.79	0.79	1.00 (0.00)	2.73 (1.37)	1.03 (0.09)	1.84 (0.47)	3
AMO	3.37 (1.91)	3.00	0.90	0.90	3.71 (1.81)	4.11 (1.86)	1.19 (0.33)	2.26 (1.02)	3
**IMI**									**12**
ENJ	5.23 (1.16)	5.43	0.86	0.87	5.01 (1.20)	4.99 (1.22)	6.07 (0.62)	5.72 (0.70)	7
TENS	3.65 (1.40)	3.60	0.81	0.82	3.48 (1.38)	4.10 (1.35)	2.81 (1.21)	3.31 (1.26)	5
**PENS**									**10**
REL	4.19 (1.63)	4.33	0.78	0.82	3.75 (1.60)	4.31 (1.69)	4.23 (1.58)	4.68 (1.38)	3
COM	5.05 (1.27)	5.00	0.79	0.80	4.98 (1.28)	4.91 (1.36)	5.37 (1.12)	5.34 (0.96)	3
AUT	4.96 (1.26)	5.00	0.75	0.76	4.72 (1.34)	4.78 (1.29)	5.67 (0.96)	5.38 (0.85)	4
**ACH_GOAL**									**11**
PerfAp	5.24 (1.58)	5.67	0.86	0.86	5.14 (1.66)	5.45 (1.52)	4.87 (1.61)	5.12 (1.47)	3
PerfAv ^3^	4.24 (1.71)	4.25	0.65	0.65	4.05 (1.79)	4.68 (1.62)	3.39 (1.62)	3.99 (1.54)	2
MastAp	4.86 (1.61)	5.00	0.82	0.82	4.65 (1.77)	4.98 (1.56)	4.85 (1.53)	4.91 (1.43)	3
MastAv	3.70 (1.78)	3.67	0.85	0.86	3.50 (1.78)	4.19 (1.77)	2.76 (1.59)	3.37 (1.5)	3
**Passion**									**10**
HP	4.06 (1.34)	4.20	0.79	0.79	3.83 (1.36)	3.98 (1.39)	4.38 (1.33)	4.46 (1.03)	5
OP	2.47 (1.42)	2.20	0.87	0.87	2.32 (1.44)	2.92 (1.48)	1.50 (0.75)	2.21 (1.04)	5
**PANAS**									**20**
PA	35.68 (7.17)	36	0.84	0.84	34.58 (7.69)	35.43 (7.13)	37.50 (6.85)	37.16 (5.96)	10
NA	22.14 (7.27)	21	0.81	0.81	21.51 (6.31)	24.98 (7.50)	16.38 (5.44)	19.68 (5.48)	10
**VITALITY**	3.59 (1.16)	3.57	0.78	0.89	3.37 (1.19)	3.55 (1.15)	3.87 (1.28)	3.92 (0.91)	**7**

#### 3.4.1. User Motivation Inventory (UMI)

To measure the six motivational regulations outlined in section 2.2, we employed the User Motivation Inventory (UMI, Brühlmann et al., 2018). The UMI is a validated 18-item questionnaire, which distinguishes amotivation, external regulation, introjected regulation, identified regulation, integrated regulation, and intrinsic motivation in the context of technology use. While all based on SDT, we chose the UMI over the SIMS (Guay et al., 2000) and ACTA (Peters et al., 2018), as they do not assess introjected and integrated regulation or amotivation, respectively. We also considered the UMI more suitable than the GAMS (Lafrenière et al., 2012). While it specifically measures motivational regulations in the context of gaming, it does not account for social aspects of (external) motivational regulation (Lafrenière et al., 2012), which we expected to be particularly pertinent to the experience of playing *LoL* with others (Tyack et al., 2016; Mora-Cantallops and Sicilia, 2018).

#### 3.4.2. Player Experience Need Satisfaction (PENS)

Psychological need satisfaction is a core concept in SDT (Deci and Ryan, 2000; Ryan and Deci, 2000), and motivational regulation is known to shape the extent to which experiences satisfy people's psychological needs of autonomy, competence, and relatedness. Need satisfaction is also prevalent in player-computer interaction research (Tyack and Mekler, 2020), where it has been consistently linked to positive player experience across a variety of genres (Ryan et al., 2006; Johnson et al., 2015, 2016) and playing persistence (Neys et al., 2014). However, with regards to MOBA games, players have reported less autonomy satisfaction, as well as increased frustration (Johnson et al., 2015), hinting at a possible relation to competence. For these reasons, we included the Player Experience Need Satisfaction scale (PENS, Ryan et al., 2006) to assess players' perceptions of autonomy, competence, and relatedness when playing *LoL*.

#### 3.4.3. Interest-Enjoyment and Pressure-Tension (IMI)

Intrinsically motivated behavior is characterized by the experience of interest and enjoyment (Deci and Ryan, 2000; Ryan and Deci, 2000). Hence, we employed the dimension interest-enjoyment of the Intrinsic Motivation Inventory (IMI, Ryan et al., 1983; McAuley et al., 1989) to assess self-reported intrinsic motivation. The IMI is commonly employed in player-computer interaction as a proxy for game enjoyment and positive player experience (Tyack and Mekler, 2020). We also included the pressure-tension dimension of the IMI, because it is a negative predictor of intrinsic motivation (Deci and Ryan, 2000; Ryan and Deci, 2000), and because it commonly characterizes the experiences of MOBA players (Johnson et al., 2015; Tyack et al., 2016).

#### 3.4.4. Positive and Negative Affect (PANAS)

Players of MOBA games, such as *LoL*, often experience pronounced positive and negative affect (Johnson et al., 2015; Tyack et al., 2016). Hence, we employed the PANAS by Watson et al. (1988) to assess positive affect (PA) and negative affect (NA). Items were rated on a 5-point Likert-type scale.

#### 3.4.5. Vitality

Mora-Cantallops and Sicilia (2018) called for more research into the impact of MOBA play on player well-being. Hence, we measured vitality, an established well-being index in SDT-based research (Ryan and Frederick, 1997). Specifically, people's experience of vitality varies as a function of both contextual and psychological factors, for instance, to the degree that one is unburdened by external pressures. We employed the vitality scale developed by Ryan and Frederick (1997). Item wording was adapted to fit the survey context, for instance, “When I play *LoL* I feel alive and vital.”

#### 3.4.6. Harmonious and Obsessive Passion

As we decided to advertise the survey on the *League of Legends* subreddit, we expected that most participants would be very passionate players of the game. However, passion to play can be harmonious or obsessive (Przybylski et al., 2009; Puerta-Cortés et al., 2017; Schaekermann et al., 2017; Perry et al., 2018). Hence, we included measures of harmonious and obsessive passion (Vallerand et al., 2003). Specifically, *harmonious passion* describes the autonomous and self-determined internalization of an activity into one's identity (Vallerand et al., 2003), whereby the activity is aligned with different areas of a person's life (i.e., they have freely chosen to play *LoL* and the activity “harmonizes” with other areas of their life, and does not interfere with their work or social life). In contrast, *obsessive passion* refers to non-self-determined internalization of an activity due to external or internal pressure (i.e., the person feels compelled to play *LoL*, for example, because of other players or personal dependencies; Vallerand et al., 2003). As such, harmonious and obsessive passion are closely linked to motivational regulation and have also been found to impact the amount of play, game enjoyment, and tension following play (Przybylski et al., 2009).

We employed an adapted version of the Harmonious and Obsessive Passion for Gambling scale (Vallerand et al., 2003; Przybylski et al., 2009). To match the context of the study, items were re-worded by replacing “this activity” with “*LoL”*.

#### 3.4.7. Achievement Goals

The gameplay of *LoL* is performative and often highly competitive in nature. Therefore, we measured players' achievement goals orientation. While not per se based on SDT, achievement goals orientation refers to how people approach competence-relevant behavior, such as studying or training (Elliot and McGregor, 2001), where different achievement goals have been found to impact intrinsic motivation to varying degrees (Chen et al., 2019). Specifically, Elliot and McGregor (2001) distinguish four related, albeit distinct achievement goals. *Mastery approach* goal orientation refers to a focus on mastering an activity and developing skills, whereas *mastery avoidance* focuses on not losing previously acquired knowledge or skills. Mastery approach, in particular, has been linked to intrinsic motivation and is associated with a wide range of positive effects in educational settings (Elliot and McGregor, 2001). In contrast, people oriented toward *performance avoidance*^3^ strive not to underperform relative to normative standards or peers, while *performance approach* is oriented toward performing better than peers or externally imposed standards. Such a performance orientation has been linked to extrinsic motivation and reduced intrinsic motivation. To measure these four orientations, we employed the achievement goal questionnaire developed by Elliot and McGregor (2001).

#### 3.4.8. Behavioral Game Metrics

Using the summoner name and region provided by participants, match histories and behavioral in-game data up until August, 16, 2018 were obtained from the API using *Riot-Watcher* (Przybylski et al., 2018)—a Python wrapper for the Riot Games API. For some matches, detailed data was not available or was incomplete. These matches were excluded from subsequent processing. We chose to focus on more recent matches played during Season 7, as well as—at the time of data sampling—ongoing Season 8 (including its preseason), i.e., matches played between January 30, 2017 and August 16, 2018. This procedure resulted in a total of 1,179,828 matches. During this period, three game maps with fundamentally different types of gameplay, strategy, match length, and team size were available (Summoner's Rift, The Twisted Treeline, and Howling Abyss). To exclude possible variability in the data due to these differences, the analysis was focused on the most popular game map, Summoner's Rift (973,564 [82.5%] of all matches). Two participants had to be excluded from the analysis because no data was available for this map.

In-game metrics for individual players derived from these matches were aggregated separately for ranked and unranked matches and, when appropriate, normalized to account for different numbers of matches.

Measures that were considered relevant for ranked and unranked matches separately include *time played, win rate, deaths, kills, assists* (i.e., helping an ally to kill an opponent), *kda* (describing the ratio of kills, deaths and assists), *killing sprees* (requiring a player to kill a certain amount of enemies without dying), *total damage dealt, total heal* (restoring one's own or an ally's health), *gold earned* (gold as in–game currency can be earned either passively (i.e., automatically without player interaction) or by actively performing certain actions, such as killing units), *gold spent* (gold can be spent on items which provide further benefits to the player) and *champions played* (the number of different champions played). Moreover, players' *level* (as a measure of experience) and *total number of matches played* represent aggregated measures over ranked and unranked matches. In total, these measures account for broad information on time, performance, and economy related in-game behavior. Note that the level of a summoner is roughly indicative of how much time a player spent playing a game and determines whether they can access some features of the game. Most prominently, a summoner level of 30 or higher is required to play ranked games. The maximum summoner's level cap was changed in the end of 2017 from 30 to limitless. The constraint of level 30 to play ranked games remained unchanged. See Table 2 for a description and Table 3 for descriptive statistics of each metric.

**Table 2:** Description of in-game measures.

**Feature**	**Description**
**COMBINED**	
totalMatches	Total number of matches (ranked and unranked)
level	The level of the summoner level
**RANKED AND UNRANKED**	
timePlayed	Total time spent in matches [in hours]
winrate	Won matches/total matches [in %]
kda	(∑ kills + ∑ deaths)/∑ assists
deaths	Avg. number of deaths per match
kills	Avg. number of kills per match
assists	Avg. number of assists per match
killingSprees	Avg. number of killing sprees per match
totalDamageDealt	Avg. total damage dealt per match
totalHeal	Avg. total heal per match
goldEarned	Avg. gold earned per match
goldSpent	Avg. gold spent per match
championsPlayed	Number of different champions played

**Table 3:** Means (M), standard deviations (SD), medians (Mdn) for behavioral metrics from the game map *Summoner's Rift* over all participants and for each profile (*N* = 748).

	**M (SD)**	**Mdn**	**Amotivated ( 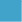 )**	**External ( 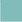 )**	**Intrinsic ( 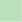 )**	**Autonomous ( 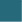 )**
			***n* = 220**	***n* = 329**	***n*** **= 89**	***n*** **= 110**
**COMBINED**						
totalMatches	1577.31 (860.47)	1455.50	1509.59 (808.3)	1596.26 (908.54)	1560.15 (828.52)	1669.96 (839.12)
level	90.75 (31.34)	88	86.31 (29.41)	92.62 (33.46)	90.38 (30.44)	94.33 (28.54)
**RANKED**						
timePlayed	292.12 (291.92)	217.50	315.39 (293.98)	283.11 (302.02)	288.79 (289.26)	275.25 (258.32)
winrate	51.32 (7.93)	51.29	51.04 (6.56)	51.54 (8.49)	51.07 (6.2)	51.45 (9.79)
kda	2.78 (0.9)	2.70	2.68 (0.62)	2.74 (0.58)	2.82 (0.63)	3.04 (1.81)
deaths	5.28 (0.99)	5.22	5.38 (0.98)	5.3 (0.98)	5.2 (1.02)	5.09 (1)
kills	5.26 (1.88)	5.53	5.31 (1.77)	5.36 (1.91)	4.99 (1.85)	5.12 (2.03)
assists	8.83 (2.37)	8.39	8.6 (2.21)	8.78 (2.42)	9.16 (2.45)	9.18 (2.39)
killingSprees	1.15 (0.46)	1.22	1.17 (0.43)	1.16 (0.46)	1.09 (0.44)	1.11 (0.5)
totalDamageDealt	108736.02 (37386.71)	118687.60	110965.31 (36541.62)	110363.24 (36371.49)	104970.8 (40501.7)	102456.99 (39010.61)
totalHeal	5577.06 (2313.61)	5133.46	5602.27 (2212.22)	5622.33 (2390.04)	5418.86 (2293.13)	5519.25 (2320.58)
goldEarned	11079.72 (1199.34)	11276.85	11136.71 (1210.19)	11137.29 (1176.31)	10970.12 (1232.92)	10882.27 (1207.63)
goldSpent	10043.92 (1167.41)	10237.11	10115.51 (1175.32)	10099.83 (1141.06)	9917.16 (1194.8)	9836.08 (1190.54)
championsPlayed	45.82 (31.52)	39.50	49.82 (34.96)	44.18 (28.46)	44.2 (30.7)	44.02 (33.29)
**UNRANKED**						
timePlayed	332.4 (281.74)	253.50	298.31 (281.84)	349.28 (283.64)	343.71 (281.26)	340.97 (273.95)
winrate	54.39 (7.32)	52.96	55.23 (9.37)	54.14 (6.31)	52.68 (4.97)	54.82 (6.87)
kda	2.69 (1.2)	2.47	2.76 (1.84)	2.65 (0.77)	2.55 (0.55)	2.78 (1.01)
deaths	6.14 (1.39)	6.07	6.21 (1.55)	6.14 (1.33)	6.17 (1.19)	5.98 (1.36)
kills	7.26 (2.24)	7.11	7.44 (2.3)	7.33 (2.25)	6.79 (2.19)	7.04 (2.07)
assists	8.25 (1.55)	8.13	8.04 (1.58)	8.19 (1.49)	8.47 (1.53)	8.64 (1.61)
killingSprees	1.54 (0.49)	1.52	1.58 (0.5)	1.55 (0.48)	1.47 (0.49)	1.51 (0.48)
totalDamageDealt	111332.13 (26068.91)	113456.90	111426.6 (26398.6)	112084.53 (24939.86)	110957.54 (29379.94)	109195.91 (26118.44)
totalHeal	5056.64 (1262.85)	4963.70	4914.68 (1225.82)	5123.63 (1234.24)	5023.89 (1276.16)	5166.66 (1394.35)
goldEarned	12075.91 (1396.49)	12053.52	12165.74 (1490.8)	12028.94 (1375.63)	12065.53 (1419.59)	12045.12 (1246.69)
goldSpent	10944.39 (1373.6)	10917.23	11042.02 (1486.24)	10895.64 (1350.68)	10912.57 (1319.88)	10920.7 (1252.36)
championsPlayed	85.65 (33.3)	88.50	84.15 (34.28)	86.36 (33.46)	84.88 (32.6)	87.13 (31.7)

## 4. Results

The results are structured as follows: First, we report correlations between self-report player experience measures and in-game metrics. Second, we test the measurement model of the UMI and use the factor scores to identify distinct motivational profiles. Third, the different motivational profiles are compared in terms of player experience and in-game behavior. Descriptive statistics for all self-report measures are presented in Table 1 and for all behavioral metrics in Table 3.

### 4.1. Correlation Analysis

To assess to what extent motivational regulation was related to participants' in-game behavior, we calculated a series of Pearson correlations. Overall, several significant correlations emerged between the different motivational regulations and in-game behavior, ranging from small to moderate. For the sake of brevity, only significant correlations with *r* ≥ |0.1| (Pearson correlation, bootstrapped *p*-values with 1,000 iterations) are reported here. Individual *p*-values and the complete correlation matrix are included as Supplementary Material.

Amotivation correlated negatively with *assists* in unranked (*r* = −0.13) and in ranked matches (*r* = −0.11) and positively with *goldSpent* in ranked matches (*r* = 0.10). Put differently, more amotivated players were less likely to assist other players in kills but spent more gold in ranked matches.

External regulation was only correlated positively with *totalHeal* unranked (*r* = 0.13). Introjected regulation, however, correlated positively with *totalMatches* (*r* = 0.10), *level* (*r* = 0.11), *timePlayed* ranked (*r* = 0.11), *winrate* ranked (*r* = 0.10), and *championsPlayed* ranked (*r* = 0.10). This suggests that players were more motivated to avoid feelings of guilt or failure, spent more time playing *LoL*, especially ranked matches. Moreover, introjected regulation was also positively correlated with *killingSprees* (*r* = 0.11), as well as ranked (*r* = 0.11) and unranked *kills* (*r* = 0.11).

For identified regulation, only two noteworthy correlations were observed: Players who considered playing *LoL* important, had played more *totalMatches* (*r* = 0.11) and achieved a higher *level* (*r* = 0.15). Similar correlational patterns emerged for integrated regulation (*r* = 0.11 and *r* = 0.14, respectively). Additionally, integrated regulation correlated positively with *timePlayed* ranked (*r* = 0.14) and *championsPlayed* ranked (*r* = 0.13).

Finally, intrinsic motivation correlated positively with achieved *level* (*r* = 0.12) and *assists* in ranked matches (*r* = 0.11). Intrinsic motivation was also negatively correlated with *kills* unranked (*r* = −0.10), *killingSprees* unranked (*r* = −0.11), *totalDamage* ranked (*r* = −0.10) *goldEarned* unranked (*r* = −0.11), *goldSpent* unranked (*r* = −0.12), *goldEarned* ranked (*r* = −0.11), and *goldSpent* unranked (*r* = −0.10). This suggests that intrinsically motivated players scored fewer kills in unranked matches, dealt less damage in ranked matches, as well as earned and spent less gold overall.

Note that correlation analysis offers only variable-centered insights into relationships between particular motivational regulations and individual metrics. Recall that SDT instead conceptualizes motivation as a multi–dimensional construct, spanning a continuum of self-determination (Deci and Ryan, 2000). Hence, it is more insightful to study how combinations of motivation variables relate to experiential and behavioral variables, rather than individual (cor)relations. Moreover, our goal was to go beyond variable-centered approaches and apply a person-centered method to identify qualitatively different motivational profiles of *LoL* players.

### 4.2. Motivational Profile Analysis

#### 4.2.1. Confirmatory Factor Analysis (CFA)

To test the measurement model of the UMI, a six-factor confirmatory factor analysis (CFA) was conducted. All items were specified to load on their designated factor, and the loading of the first item was constrained to one. Multivariate normality was not given (Mardia tests: χ^2^ = 4644.83, *p* < 0.001, *Z*_*k*_ = 52.98, *p* < 0.001), hence a robust Maximum Likelihood Estimation method with Huber-White standard errors and a Yuan-Bentler based scaled test statistic was used^4^. Results of the CFA suggested that the six factor model fits the data well [χ^2^ = 257.21, *p* < 0.001, χ^2^/*df* = 2.14, *CFI* = 0.972, SRMR = 0.050, RMSEA = 0.039, PCLOSE = 0.999].

#### 4.2.2. Latent Profile Analysis (LPA)

Latent Profile Analysis (LPA) is a latent variable modeling technique that detects clusters of observations with similar values on cluster indicators (Pastor et al., 2007). In other words, it can be used to identify combinations of motivation variables, which can then be related to other variables, such as player experience and in-game behavior, while circumventing the aforementioned issues around correlation analysis. Although a relatively novel technique, it has previously been applied in SDT research to study motivation in educational (Pastor et al., 2007; Wang et al., 2017), work (Howard et al., 2016) and athletic settings (Gustafsson et al., 2018).

To assess whether the data exhibited distinct motivational profiles, we conducted an LPA using factor scores retained from the CFA six factor model. Conducting an LPA with factor scores instead of scale scores allows for partial control of measurement errors by giving more weight to items (Howard et al., 2016; Kam et al., 2016). When determining the optimal number of profiles, it is key to consider not only the statistical adequacy of the found solution, but also the theoretical conformity of the profiles (Morin and Marsh, 2015; Howard et al., 2016). In deciding upon our final model, information-based methods like the Bayesian Information Criterion (BIC) and Integrated Complete-data Likelihood (ICL), as well as resampling methods, such as the Bootstrap Likelihood Ratio Test (BLRT), were considered for each solution (Scrucca et al., 2016). Other indices, such as entropy, AIC, LMR, ALMR are not recommended for selecting the optimal number of profiles (Tofighi and Enders, 2008; Diallo et al., 2017).

The estimated fit indices proposed a divergent optimal number of profiles. The BIC, ICL, and investigation of the Elbow plots indicated that four profiles were most appropriate and parsimonious (BIC (VVV), five groups: −10652.0, ICL (VVV), four groups: −10771.4). Visual interpretation of the elbow plot for the BIC criterion also revealed four groups to be most appropriate. In contrast, the BLRT found the optimal group size to be seven, reflecting the data (Likelihood Ratio Test 7 vs. 8 groups: −165.92, *p* = 0.996). After considering the theoretical conformity of the profiles (i.e., resulting group sizes, group specific motivational profiles), we deemed four profiles to be optimal.

**Figure 2:**
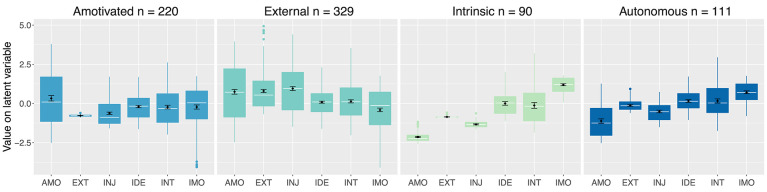
Motivational pattern of the four profiles identified in the sample. The white lines in the boxplot indicate the median and the black rhombi indicate the mean with bootstraped 95% confidence intervals (1,000 iterations).

Figure 2 shows the distribution of scores for all six motivational regulations for each of the four profiles, where 0.0 depicts the overall mean score for each latent variable (i.e., *M* = 5.31 for intrinsic motivation; *M* = 3.08 for integrated regulation, etc.). As listed in Table 1, participants overall reported high levels of intrinsic motivation (*M* = 5.31, *SD* = 1.39) and low scores on the remaining regulations, especially introjected (*M* = 2.35, *SD* = 1.55) and external regulation (*M* = 1.88, *SD* = 1.22).

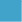
 Profile 1 (*n* = 220) was characterized by above average amotivation. Compared to other players, participants in this profile also reported below average intrinsic motivation and external regulation, while the other motivational regulations scored close to 0.0 (i.e., average). This does not mean that this player profile lacked in intrinsic motivation. In fact, players in this profile reported considerable intrinsic motivation (*M* = 5.11, see Table 1). However, participants' rather elevated amotivation ratings (*M* = 3.71, Table 1) were what primarily differentiated Profile 1 from the other profiles. Based on the motivational spectrum posited by SDT (see Figure 1), we hence refer to Profile 1 as “Amotivated.”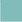 Profile 2 (*n* = 329) featured markedly above average scores on amotivation, external and introjected regulation, as well as slightly above average scores on identified regulation and integrated regulation. While still considerable (*M* = 4.97), intrinsic motivation scores were below average, compared to the overall sample. Similar to the “Amotivated” profile, players in this profile reported considerable amotivation (*M* = 4.11). However, what distinguishes Profile 2 from the other profiles, are the comparably higher scores on external and introjected regulation (*M* = 2.73 and *M* = 3.33, respectively). Hence, we dubbed this the “External” profile.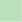 Profile 3 (*n* = 90) scored above average on intrinsic motivation, whereas the other motivational regulations were at average or below average levels. In other words, players in this profile were predominantly intrinsically motivated, and accordingly scored high on intrinsic motivation (*M* = 6.34). Hence, we refer to this as the “Intrinsic” profile.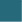 Profile 4 (*n* = 111) scored above average on intrinsic motivation (*M* = 5.92), but less so than the “Intrinsic” profile. Moreover, it featured slightly above average levels on identified and integrated regulation, as well as average levels of external regulation. In contrast to the “Intrinsic” profile, players in this profile were most characterized by a blend of intrinsic motivation and slightly higher scores on the other motivational regulations. Nevertheless, as the “autonomous” regulations (i.e., intrinsic motivation, identified and integrated regulation, Deci and Ryan, 2000; Ryan and Deci, 2000) were more salient, we refer to this as the “Autonomous” profile.

### 4.3. Player Experience

**Figure 3:**
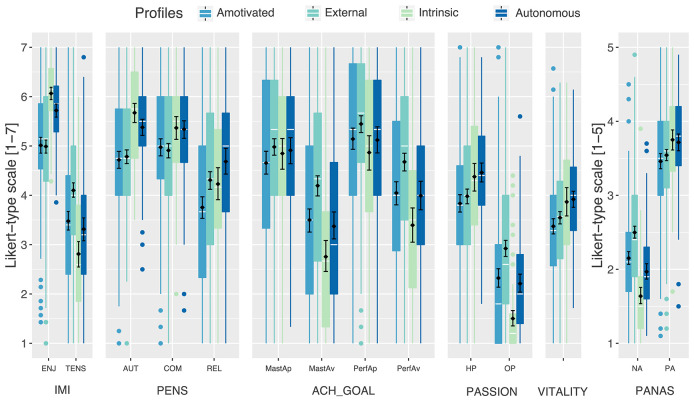
Distribution of the values on the different player experience measures. The white lines in the box plot indicate the median and the black rhombi indicate the mean with bootstraped 95% confidence intervals (1,000 iterations).

Kruskal-Wallis rank sum tests were conducted to test whether the four motivational profiles differed significantly with regards to the self-report player experience measures. Statistically significant differences were found for every measure at an alpha-level of .001. However, due to the exploratory nature of this study and the large number of variables, the results are interpreted based on descriptive statistics (means, medians, and distributions). Note also that statistical significance testing between each pair of profiles for all measures would greatly increase the likelihood of type 1 errors (i.e., false positives). Therefore, Figures 2, 3 include a bootstrapped (1,000 iterations) 95% confidence interval of the mean. If the proportion of overlap of 95% confidence intervals of two means is 0.5 or less, they indicate statistical significance at an alpha-level of 5% (Cumming and Finch, 2005).

As pictured in Figure 3 (see also Table 1), all profiles reported high enjoyment, especially the Intrinsic player profile (*M* = 6.07, *SD* = 0.62). In contrast, the External profile scored highest on tension. Moreover, all motivational profiles scored relatively high on relatedness, autonomy, and competence need satisfaction, with relatedness being least salient. However, the Intrinsic and Autonomous player profiles reported the highest levels of need satisfaction for all three needs, where the latter scored highest on relatedness.

With regards to achievement goals, participants overall scored highest on performance approach, followed by mastery approach and performance avoidance. Looking at the individual profiles, the External player profile reported the highest levels of performance approach and avoidance, as well as mastery avoidance. In contrast, the Intrinsic profile scored lowest on avoidance for both performance and mastery. Mastery approach was comparable between profiles, but lowest for Amotivated players.

In general, participants scored low on obsessive passion and around midpoint (*M* = 4.06) on harmonious passion. The Autonomous and Intrinsic player profiles reported the highest levels of harmonious passion, with the Intrinsic profile scoring particularly low on obsessive passion. In contrast, External players reported markedly higher levels of obsessive passion compared to the other profiles.

Overall, vitality after playing *LoL* was slightly below midpoint (*M* = 3.59, *SD* = 1.16), where the Autonomous and Intrinsic profiles experienced more vitality than the Amotivated and External players.

Finally, with regards to affect, the Amotivated and especially the External profiles reported markedly increased levels of negative affect compared to the other profiles. Positive affect was rather pronounced for all profiles, but more so for the Autonomous and Intrinsic player profiles.

### 4.4. Behavioral Game Metrics

**Figure 4:**
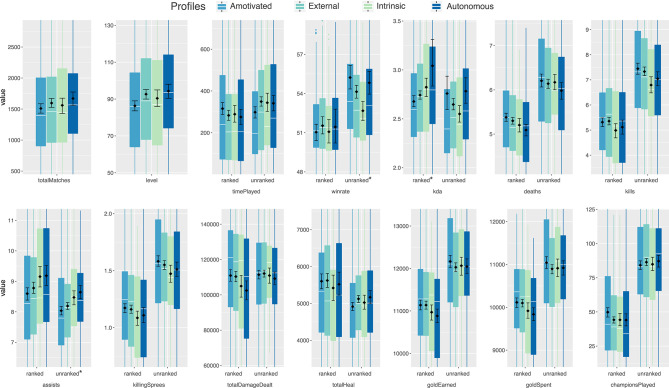
Comparison of behavioral metrics between the four profiles. The white lines in the box plot indicate the median and the black rhombi indicate the mean with bootstraped 95% confidence intervals (1,000 iterations). Asterisks highlight statistically significant differences with Kruskal-Wallis rank sum tests and α = 0.05.

An overview of all behavioral metrics is presented in Table 3, and Figure 4 includes confidence intervals for the means. Overall, participants had played almost 1,600 matches on average between January 30, 2017, and August 16, 2018. More time was spent playing unranked than ranked matches. In the following, each metric will be compared between the four profiles. A series of Kruskal-Wallis rank sum tests was conducted to test whether there were overall significant differences in the behavioral data. Results showed that *winrate* unranked, χ^2^(3) = 9.68 *p* < 0.05, *kda* ranked, χ^2^(3) = 10.9 *p* < 0.05, and *assists* unranked, χ^2^(3) = 14.64 *p* < 0.05, showed significant differences between profiles.

#### 4.4.1. Number of Matches, Level, and Playtime

For the total amount of matches and the average level of the players, a slight increase from the Amotivated toward the Autonomous player profile is visible. Amotivated players spent the most time playing ranked matches and the least amount of time in unranked matches. These players seem to be more ranked games oriented. However, they were on average on a lower in-game level, whereas the Autonomous profile featured more higher-level players.

#### 4.4.2. Performance Measures

With players being keen on improving their performance, as shown by the high scores on performance approach orientation, we were interested in exploring the relations between wins and losses, as well as kills, deaths, and assists.

For unranked matches, Amotivated players showed a significantly higher *winrate* than the Intrinsic player profile (*Z* = 2.923, *p* < 0.05, Dunn's multiple comparison with *p*-values adjusted with the Holm method), while the Autonomous and External profiles are in-between. In ranked matches, a comparison of the *winrate* reveals very similar means for all profiles, slightly above 50% each, confirming the effectiveness of the *LoL* match-making mechanism.

However, in terms of the number of deaths in ranked matches, the more self-determined profiles “Intrinsic” and “Autonomous” show lower values, but they also score less kills in both ranked and unranked matches. Intrinsic and Autonomous players scored more assists in ranked and unranked matches. For unranked matches, *post-hoc* comparisons showed that Autonomous and Intrinsic player profiles performed statistically significant more assists than Amotivated profile (*Z* = 3.224, *p* < 0.05; *Z* = 2.794, *p* < 0.05).

The kill-death-assist ratio (*kda*) in ranked matches suggests that Autonomous players were the highest-performing profile, whereas the Amotivated profile performed worst (*Z* = 2.922, *p* < 0.05). Descriptively, the pattern is less clear for unranked matches where intrinsically motivated players have the lowest average value and amotivated and autonomous players are on par. However, the differences between the mean and median values is relatively large, suggesting that there are a outliers present who have very high *kda* values in unranked matches.

Taken together, the Amotivated profile's champions die the most, but they also kill more opponents compared to both Intrinsic and Autonomous player profiles. This may suggest that Amotivated players exhibit a more “reckless” playstyle compared to other profiles. However, this behavior appears less successful in ranked matches than in non-ranked ones, as indicated by the *kda* ratio and the *winrate*.

#### 4.4.3. Economy Related Behaviors

Across all profiles the amount of gold earned and spent in both ranked and unranked matches is very similar, with only ranked matches showing slight differences. With multiple sources and ways to acquire gold, it is however difficult to determine how the motivational profiles relate to gold earned.

## 5. Discussion

Playing games is commonly considered an enjoyable and intrinsically motivating activity (Ryan et al., 2006; Przybylski et al., 2010). League of Legends and other MOBA games, however, are massively popular, despite players reporting comparatively subpar experiences relative to other game genres (Johnson et al., 2015). The present study shows that people's underlying motivational regulations for playing *LoL* may play a crucial role therein. Based on Organismic Integration Theory, a mini-theory of Self-Determination Theory (Deci and Ryan, 2000), we identified four distinct motivational profiles, which differed markedly in their player experience and, to a lesser extent, in their in-game behavior. The Intrinsic player profile reported overall the most positive experience. Contrary to previous findings that MOBA games afford less autonomy and more frustration than other game genres (Johnson et al., 2015), players in this profile experienced a considerable sense of autonomy and competence when playing LoL, as well as reported low levels of tension and negative affect. In contrast, the Amotivated and External player profiles seem to derive markedly less enjoyment from their playing experience, as well as reported more tension and negative affect. They also scored lower on experienced autonomy and competence need satisfaction—with autonomy ratings similar to the ones reported by Johnson et al. (2015) (i.e., below *M* = 5.0).

As such, our findings are in line with OIT and previous research on motivational regulations and technology use. As posited by SDT, more self-determined player profiles (i.e., Intrinsic and Autonomous profiles) reported a more positive experiences (Deci and Ryan, 2000) and more harmonious passion for play (Vallerand et al., 2003), compared to the less self-determined profiles (Amotivated and External profiles). Moreover, recall that previous research found people reporting higher levels of amotivation to be more at risk of burn out (Gustafsson et al., 2018), as well as more likely to consider abandoning a technology (Brühlmann et al., 2018). As such, players in the Amotivated profile might be more inclined to quit playing LoL. While participants in our sample may be considered dedicated players, as evidenced by their being active in the *LoL* subreddit, the Amotivated and External profiles enjoyed playing substantially less. Indeed, lack of fun is one of the reasons players stop engaging with MOBAs (Tyack et al., 2016).

Our results also support existing findings on motivation and achievement goal orientation (Elliot and McGregor, 2001; Chen et al., 2019). Compared to the other profiles, the External profile scored higher on performance approach and performance avoidance orientation. Recall that this profile is more motivated by external pressure and avoiding feelings of guilt. These players may therefore feel particularly driven to perform well in *LoL* relative to their peers. However, performance and mastery approach orientation was rather high across all profiles, which is not surprising, considering the highly competitive nature of *LoL*, where players strive to improve their skills and perform well in front of their teammates (Johnson et al., 2015; Kahn et al., 2015; Tyack et al., 2016; Mora-Cantallops and Sicilia, 2018).

Next, the four profiles differ considerably in group size. Many more players fell into the Amotivated (29.3%) and External (43.9%) profiles than the Intrinsic (12%) or Autonomous profiles (14.8%). As such, it seems that a majority of players have a less positive experience when playing *LoL* and are not purely driven by intrinsic motivation. While Johnson et al. (2015) did not recruit participants over Reddit, it could be that the majority of MOBA players in their sample also fell into the Amotivated or External profiles, which might explain their more negative player experience ratings. What is less obvious is why these players reported less self-determined motivations. As of now, it is unclear if these players were already more amotivated and/or externally motivated when they started playing *LoL*—perhaps not to let a friend down Tyack et al. (2016),—or whether their motivation shifted over time.

Notably, our group size numbers are inconsistent with previous work on motivational regulation profiles. In their study of elite athletes, Gustafsson et al. (2018) found that only 22% of participants fell into the amotivated and moderately controlled profile (i.e., they reported more external and introjected regulation), with even fewer falling into the predominantly amotivated profile (6.9%). Similarly, in a study on work motivation (Howard et al., 2016), between 13.1 and 27.6% of participants were classified into the amotivated profile. With regards to the Intrinsic and Autonomous profiles, our findings are more comparable. The autonomous profiles in the aforementioned studies (Howard et al., 2016; Gustafsson et al., 2018) encompassed 15.9–25.6% of all participants.

Importantly, our study showcases that participants' motivations for playing *LoL* are not mutually exclusive. While some motivations were more salient for certain profiles (e.g., the Intrinsic profile), most profiles can be considered a motivational blend, where intrinsic motivation was reported along amotivation and other motivational regulations. Indeed, profiles share some considerable overlap, as intrinsic motivation was rather high across all player profiles. This is not surprising, as intrinsic motivation (i.e., seeking enjoyment in an activity) and the experience of enjoyment are key motivators for play for casual, heavy, and hardcore gamers (Neys et al., 2014).

In contrast to previous work on motivational profiles (Howard et al., 2016; Gustafsson et al., 2018), we observed no “high” motivation profile, i.e., where people score high on all motivational regulations, except amotivation. At least with regards to highly involved *LoL* players (i.e., active on the subreddit), it seems that certain motivational regulations are more salient (e.g., amotivation, intrinsic motivation). Nevertheless, our findings suggest that even small increments in amotivation, external and identified regulation are already associated with a less positive experience (operationalized as increased enjoyment, positive affect and need satisfaction, as well as lower levels of tension and negative affect).

### 5.1. Motivation and In-Game Behavior

Results indicate that motivational regulations shape patterns of need satisfaction and player experience. However, the four player profiles exhibited fairly similar in-game behavior overall. We found several statistically significant, albeit small to moderate correlations between game metrics and self-report measures. These results indicate a slight linear relationships between certain behaviors and motivational regulation. This is not surprising, as previous research examining game metrics and self-reported experience measures also reported low to medium correlations (Canossa et al., 2013; Schaekermann et al., 2017; Melhart et al., 2019). Among the 14 metrics we studied, the four motivational profiles varied significantly in terms of their *winrate* and assists in unranked matches, and kill-death-assist ratios in ranked matches (see also Table 3 and Figure 4).

For kill-death-assist ratios in ranked matches and assists in unranked matches, the Amotivated profile showed the lowest median sore, while the more self-determined player profiles show slightly higher performance, especially in unranked matches.

In unranked matches, the Intrinsic player profile was characterized by an increased number of assists and a low rate of won games on the Summoner's Rift map. However, this profile did not report higher levels of relatedness. Hence, it would be misleading to claim that this profile featured more social or supportive players. Rather they seem to perceive their game play as highly autonomous and experience the most enjoyment of all profiles. Thus, they may simply enjoy the game and care less about winning than the other players, as reflected by the lower scores on performance approach and avoidance goal orientations.

However, behavioral metrics collected in this study are on a relatively high level of analysis (i.e., aggregated over all matches of a player) and findings need to be taken with a grain of salt. Consider that the behavioral metrics in our sample constitute of data aggregated over a longer period of time, whereas the self-report survey only covers a single measuring point. We examined metrics of *LoL* which reflect performance (e.g., winrate, kill-death-assist ratio), playstyle (e.g., killingSprees, totalHeal, championsPlayed), and engagement (e.g., totalMatches, timePlayed) aggregated over a period of about 18 months. If the effects of motivational regulations change over time, behavioral differences between the four motivational profiles may be only observable with detailed trend analyses. Further, the interplay of experience and behavior may be highly game-specific; there may be only a limited number of ways a game can be played. However, the few observed behavioral differences between the profiles show that similar behavior—with different underlying motivational regulations—can lead to very different experiences.

### 5.2. Limitations and Future Work

The present study is the first to apply OIT to better understand the interplay of player motivation, experience and in-game behavior in League of Legends. Specifically, we employed Latent Profile Analysis, a novel approach to profile players according to their motivational regulations. That said, our study comes with several caveats and limitations. First, note that due to the LPA approach, differences between profiles are relative. For example, while participants in the External player profile reported higher tension (*M* = 4.10), this is only slightly above the scale midpoint (3.5). Similarly, in terms of obsessive passion and negative affect, all profiles scored below the scale midpoint on average (3.5 and 2.5, respectively). Overall, participants in our sample did not report negative experiences when playing League of Legends. Nevertheless, it seems that minor fluctuations in motivational regulations may already shape the player experience toward more adverse or more positive outcomes.

That said, the exploratory nature of this study does not allow for causal inferences. Although in line with SDT propositions, it is unclear whether motivational regulations shape experiential outcomes and in-game behavior, whether players' experiences and behaviors impact their motivation, or—most likely—whether there are bidirectional effects. Repeated data collection of self-reported and logged behavioral data may provide more insights into how different motivational regulations affect experience and changes in behavior. It may also help mitigate certain limitations inherent to retrospective self-reports (i.e., recall bias) (Solhan et al., 2009).

Second, due to the cross-sectional design of the study (i.e., only one measuring point for self-reported motivation and experience), the present work cannot make any statements about potential changes in motivation over time. Longitudinal studies are necessary to assess whether motivational player profiles remain fairly stable, or fluctuate when players start playing, have been playing for a long time already, or decide to stop playing (Tyack et al., 2016). As such, future work should consider how long players have already engaged with *LoL* or other MOBAs.

Another promising avenue for studying motivational shifts over time is to consider the notion of internalization. Recall that SDT posits motivational regulations may shift through the process of internalization, along the controlled-to-autonomous continuum (Deci and Ryan, 2000, see also Figure 2, from left to right). When people take up values, attitudes, or regulatory structures, initially externally regulated behaviors may become internalized and then no longer require the presence of rewards or pressure (Deci and Ryan, 2000). For instance, it could be that certain players are initially both intrinsically and externally motivated. That is, they might choose to play *LoL* to experience enjoyment, but also due to perceived pressure from friends and teammates (Tyack et al., 2016). Over time, and over repeatedly experiencing a sense of autonomy, competence and relatedness, players might shift toward the Autonomous player profile, because playing *LoL* becomes personally meaningful to them. Or they might perhaps shift to the predominantly Intrinsic player profile, as they no longer feel pressured from others or themselves to play.

Longitudinal studies on players' motivational regulations could also provide insights into other aspects of MOBA play. For instance, whether professional esports athletes go through different motivational shifts than more casual players, due to experiencing more pressure to play or succeed (Deterding, 2016; Peters et al., 2018). Or whether the experience of toxic social interactions (Kwak and Blackburn, 2014; Shores et al., 2014) result in initially intrinsically motivated players shifting toward external regulation or even amotivation. Identifying such contributing factors could facilitate the design of interventions to counteract negative effects early on, as well as inform game design to promote mastery over performance orientation in players.

Third, note that the motivational profiles outlined in the present study only represent a momentary snapshot, whereas the processed behavioral data extend over a period of about 18 months—over which League of Legends has undergone several patches and changes. As such, the collected data operate on two different levels of analysis. While rather challenging and time-consuming, it would be useful to collect self-reports of motivational regulation and player experience after each season or pre-season, or better yet, after individual matches. This would allow for a more tightly coupled and granular analysis of the interplay of motivational regulation and in-game behavior, as well as help control for various changes due to patch updates and the introduction of new champions (Mora-Cantallops and Sicilia, 2018). It would also be interesting to classify players based on their in-game behavior (e.g., as in Melhart et al., 2019), and then compare them in terms of their motivational regulations.

Fourth, a sample selection bias toward highly engaged players is likely, as participants were recruited from the LoL subreddit. As such, participants were not only eager LoL players, but clearly also invested in the metagame (Donaldson, 2017), e.g., they read patch notes or discuss strategies with other players. Future studies should therefore take into account whether participants identify as hardcore or more casual *LoL* players (Neys et al., 2014), as well as how they perceive their reputation within the player community, which may affect their motivational regulation (and vice versa). Conversely, novice players might be more oriented toward mastering the game mechanics, especially when playing with friends (Tyack et al., 2016), and may not yet be as performance oriented (Elliot and McGregor, 2001).

Moreover, our sample is biased toward men, with only slightly over 6% of participants identifying as women or non-binary, slightly less than the expected 10%^5^. As gender stereotypes are known to affect the in-game character design, players' perception of abilities, and social conventions in LoL (Gao et al., 2017), future studies should be mindful of the experiences and motivations of female, non-binary and trans players.

It remains to be seen whether the present findings generalize to other MOBAs or game genres. According to SDT, the negative effects of less self-determined motivational regulations and amotivation on well-being are largely context-independent (Deci and Ryan, 2000). Hence, we expect that similar player profiles broadly manifest for other MOBAs and genres, and that motivational regulations may similarly shape players' experience—although the number and specific patterns of motivational profiles may vary to some extent.

Lastly, it would be interesting to combine OIT with other motivational frameworks or personality models. Indeed, recent works successfully combined game analytics and self-report questionnaires of player typologies to profile players and identify game design improvements (Yee et al., 2012; Canossa et al., 2013; Kahn et al., 2015; Schaekermann et al., 2017). According to SDT (Deci and Ryan, 2000), all of these motivational typologies describe “what” activity players seek to pursue (e.g., curiosity, competition, socializing, etc.), whereas the motivational regulations posited by OIT refer to the underlying reasons “why” these activities are being pursued. Similarly, according to causality orientation—another SDT mini-theory—people differ in the extent to which they generally experience their actions as self-determined (Deci and Ryan, 2000). As such, it is possible that some participants in our sample were broadly more Autonomy or Control oriented (i.e., more inclined toward autonomous or external regulations, respectively), or tended toward amotivation, regardless of any situational factors. Finally, OIT could be combined with other personality models, such as the Big Five model (Sheldon and Prentice, 2019), which has already been successfully combined with game analytics (Canossa et al., 2015).

## 6. Conclusion

We present findings from a theory-driven exploratory approach toward understanding player motivation and experiences in League of Legends. Combining Self-Determination Theory, Latent Profile Analysis and game analytics, we identified four motivational profiles, which differ with regards to player experience and, to a lesser extent, player behavior. In particular, our findings highlight the importance of considering amotivation and extrinsic regulation types, which hitherto have received only scant attention in player experience research. As such, this paper provides researchers and game designers with a novel and theoretically grounded perspective on player motivation.

## Data Availability Statement

The anonymized survey data can be accessed on the Open Science Framework https://osf.io/ue82s/. Anonymized aggregated behavioral metrics are available upon request.

## Ethics Statement

Ethical review and approval was not required for the study on human participants in accordance with the local legislation and institutional requirements. The patients/participants provided their written informed consent to participate in this study.

## Author Contributions

FB, GW, SK, and EM contributed the conception and design of the study. PB prepared the survey. EM distributed and collected the survey data. GW matched and prepared the behavioral data. FB and PB performed the statistical analysis. FB, PB, and EM wrote the first draft of the manuscript. FB, PB, GW, SK, and EM wrote the sections of the manuscript. All authors contributed to the article and approved the submitted version.

## Conflict of Interest

The authors declare that the research was conducted in the absence of any commercial or financial relationships that could be construed as a potential conflict of interest.

## Footnotes

^1^https://www.statista.com/statistics/505613/leading-digital-pc-games-by-global-revenue/ (viewed: 28. Jan 2020).

^2^100 million monthly active users in 2016 https://www.statista.com/statistics/317099/number-lol-registered-users-worldwide/ (viewed: 28. Jan 2020) and one of the free-to-play games that generated the most revenue in 2019 https://www.statista.com/statistics/346515/leading-f2p-mmo-games/ (viewed: 28. Jan 2020).

^3^*Note*. Due to an error in the survey, the item “I just want to avoid doing poorly in LoL” had to be excluded from analysis.

^4^*Note*. We also conducted an Exploratory Factor Analysis (EFA) and parallel analysis, which proposed a five-factor model instead. However, a subsequent CFA indicated that the five-factor model had a significantly worse fit (χ^2^ diff. = 84.96, *p* < 0.001).

^5^https://www.statista.com/statistics/694381/gamer-share-world-genre-and-gender/ (viewed: 28 Jan 2020).

## Abbreviations

MOBA: Multiplayer Online Battle Arena
LoL: League of Legends
SDT: Self-Determination Theory
OIT: Organismic Integration Theory
EXT: external regulation
INT: introjected regulation
IDE: identified regulation
INT: integrated regulation
UMI: User Motivation Inventory
IMI: Intrinsic Motivation Inventory
PENS: Player Experience Need Satisfaction
PANAS: Positive and Negative Affect Schedule.
